# Synbiotic intervention reverses alcohol drinking-induced cognitive deficits in adolescent male mice by modulating the microbiota-gut-brain axis

**DOI:** 10.1080/19490976.2025.2551104

**Published:** 2025-09-01

**Authors:** Marta Barrera-Conde, Elizaveta Korchevaya, Elk Kossatz, Emma Veza, Mitona Pujadas, Élida Alechaga, Pau Nebot, Oscar J. Pozo, Rafael de la Torre, Nieves Pizarro, Patricia Robledo

**Affiliations:** a Integrative Pharmacology and Systems Neuroscience Research Group, Neuroscience Research Programme, Hospital del Mar Medical Research Institute, Barcelona, Spain; b Department of Medicine and Life Sciences, University Pompeu Fabra, Barcelona, Spain; c Research Programme on Biomedical Informatics (GRIB), Hospital del Mar Medical Research Institute, Barcelona, Spain; d CIBER de Fisiopatología de la Obesidad y Nutrición (CIBERON), Instituto de Salud Carlos III, Madrid, Spain; e Applied Metabolomics Research Group, Neuroscience Research Programme, Hospital del Mar Medical Research Institute, Barcelona, Spain

**Keywords:** Adolescence, intermittent alcohol drinking, gut microbiota, behavior, short-chain fatty acids, brain glutamate, β-hydroxybutyrate

## Abstract

Adolescence is characterized by an increased vulnerability to substance abuse, including alcohol consumption. We investigated the effects of a synbiotic intervention on disruptions of the microbiota-gut-brain axis induced by a drinking in the dark model of intermittent alcohol exposure in adolescent mice. We found that alcohol drinking induced specific shifts in gut microbiota, namely it increased Erysipelotrichaceae and reduced fecal butyric and isovaleric acids. In adulthood, other types of gut bacteria were affected such as *Rhodospirillales uncultured family* and *Entrorhabdus uncultured bacterium*. Social and nonsocial cognitive impairments were also observed, and disruptions in prefrontal cortex β-hydroxybutyrate and glutamate metabolic profile in the hippocampus were apparent. Importantly, the synbiotic restored gut microbiota alterations and exerted beneficial effects on alcohol-induced behavioral impairments and brain metabolite changes. In correlational studies, we identified two potential functional networks, one relating gut microbiota (*Actinobacteria and Lactobacillaceae*)-isovaleric acid with prefrontal glutamate metabolism and sociability, and the other relating SCFAs (propionic, butyric, valeric and isovaleric acids) with β-hydroxybutyrate in the hippocampus and reference memory. These results provide correlative data showing that synbiotic supplementation may restore delayed behavioral alterations induced by voluntary sub-binge alcohol drinking during adolescence through microbiota-gut-brain interactions, and might represent a potential therapeutic tool against long-term alcohol induced behavioral and molecular disturbances.

## GRAPHICAL ABSTRACT

**Figure:**
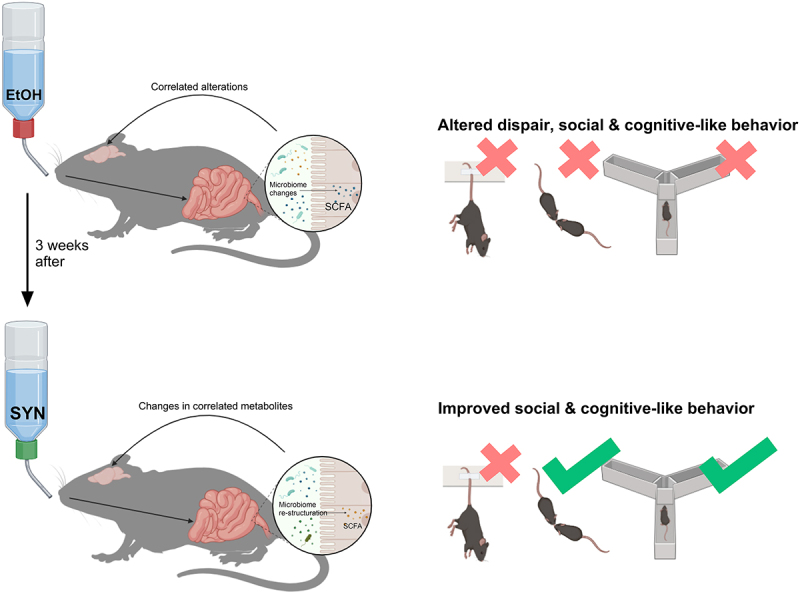

## Introduction

Adolescence is a transitional phase between childhood and adulthood, marked by significant physiological, cognitive, and social development. During this period, the brain undergoes maturation, reaching full development around the age of 25.^1,2^ Adolescents are often described as impulsive and prone to risk-taking. Furthermore, through social contacts, adolescence is a period of identity building and increasing independence from parental figures.^3^ However, this phase is also characterized by heightened vulnerability to alterations in brain maturation during encounters with harmful substances like alcohol.^4^

Alcohol drinking is a global health concern, with Europe contributing the most to the worldwide proportion of 25% of adolescent drinkers.^5^ For instance, in Spain the average age to initiate alcohol consumption is 14 years^6^, thus co-occurring with adolescence. Nowadays, young people consume huge amounts of alcohol in a short period of time, a pattern known as binge drinking. According to the NIAAA definition, a pattern of alcohol consumption that raises blood alcohol concentration (BAC) to 0.08 g/dL or above, which typically corresponds to consuming 5 or more standard drinks for males, or 4 or more for females, in approximately 2 hours can be considered as binge drinking behavior. This corresponds to 4–5 drinks for 14–17-year-old boys and more than 3 drinks for girls of the same age taken in 2 hours.^7^ Coincidentally, such activities are associated with nightlife culture, disrupting the sleep-wake cycle^8^. Moreover, alcohol drinking during adolescence can induce both long-term and delayed behavioral alterations such as social and nonsocial cognitive, and emotional impairments as evidenced in human studies and animal models.^4,9^ In addition, brain changes have been extensively reported in both rodent and human adolescents following binge alcohol drinking, including decreases in gray matter volume in the prefrontal cortex (PFC), the hippocampus (HPC), altered hippocampal neurogenesis, and disruptions in dopaminergic, glutamatergic and GABAergic neurotransmission.^10^ Moreover, an early age of first alcohol intake, as well as adolescent heavy drinking, are a risk factor for alcohol disorders in adulthood.^11,12^

Alcohol consumption can lead to an imbalance in gut bacteria, which results in enhanced intestinal permeability, inflammation, and modified immune reactions,^13,14^ known as gut dysbiosis. Additionally, it may cause enduring changes in gut microbiota in adult rats following intermittent alcohol exposure during adolescence.^15^ Interestingly, studies in humans reported that gut microbiota changes are linked to behavioral alterations in patients with alcohol use disorder^16^, and transplantation of dysbiotic microbiota to mice, recapitulates the emotional and social impairments seen in patients, which were associated with low plasma concentrations of β-hydroxybutyrate (BHB)^17^. Moreover, reduced alcohol craving and intake were mimicked in germ-free mice following fecal transplantation from recovered alcohol use disorder patients who had previously received fecal transplant therapy.^18,19^ Concurrently, an increasing amount of evidence points to a bidirectional relationship between the gut microbiota and the central nervous system through the gut microbiota-brain-axis. Thus, previous results showed that gut dysbiosis can lead to cognitive, social and emotional disorders in rodents.^20–22^

Altogether, these studies suggest that the gut microbiota can be a target to improve the host’s health through dietary supplementation with prebiotics, probiotics or synbiotic (SYN), a mixture of both.^23,24^ Multiple evidence reported that the use of probiotics reduced alcohol-related inflammation, restored the gut permeability, improved liver function and detoxification in humans.^14^ Moreover, we have recently shown that SYN treatment can alleviate some behavioral alterations caused by chronic alcohol intake in adult mice.^25^ Yet, there is lack of evidence on whether SYN administration can revert the detrimental consequences of binge-like alcohol drinking in adolescence.

Therefore, this study aimed to investigate whether a SYN supplementation could be beneficial to recover alterations in behavior and gut microbiota caused by alcohol drinking during adolescence. For this purpose, we used a mouse model of drinking in the dark (DID), where adolescent mice consume alcohol during several weeks in an intermittent alcohol drinking pattern (access to alcohol only on certain days of the week and in a short period of time),^26,27^ which has been widely adopted in preclinical research as a model for voluntary ethanol consumption that approximates binge-like intake. To understand the associated mechanisms involved in the effects of alcohol and SYN, we performed a 16S rRNA metagenomic analysis to study gut microbiota species composition and diversity. In addition, intestinal bacteria can synthesize short-chain fatty acids (SCFA)^28,29^ and modulation of these compounds within the microbiota-gut-brain axis may play a role in cognition, mood and sociability processes.^30–32^ Thus, we also determined the fecal concentrations of SCFA, and quantified brain metabolites in the PFC and HPC. Our results show that alcohol consumption during adolescence alters gut microbiota composition and impairs both social and nonsocial cognition. Although microbiota alterations were restored three weeks after alcohol exposure, specific changes in PFC and HPC metabolites were apparent as signatures of alcohol-induced damage. Finally, correlational studies indicate that SYN administration attenuates social and nonsocial cognitive deficits induced by voluntary sub-binge alcohol drinking and restores brain metabolite concentrations, suggesting that targeting gut dysbiosis could serve as a potential therapeutic approach for alcohol-related cognitive impairments.

## Materials and methods

### Animals and drinking in the dark procedure

A total of 40 C57BL/6J adolescent male mice at postnatal day (PD) 30 were randomly assigned to 2 experimental groups. One group received water (H2O; *n* = 20) and the other group received a 20% v/v alcohol solution prepared from 96% ethanol (EtOH, *n* = 20). Mice were housed in standard Plexiglas cages under controlled temperature (22 ºC) with a 12-h reversed light/dark cycle (lights off at 7:00 a.m.). In the EtOH group, adolescent mice were exposed to a modified 4-day drinking in the dark (DID) protocol over 4 weeks (PD30–57).^26,27^ Each week, on days 1–4 mice were weighed at 8:00 a.m. Three hours into the dark cycle (10:00 a.m.), mice were individually placed in a cage with a single bottle of 10 ml tap water (H2O group) or a single bottle of 10 ml alcohol (EtOH group). On the first three days of the experiment, mice had free access to H2O or EtOH for 2 h, and on the fourth day alcohol access was increased up to 4 h. During the light period mice were left undisturbed. The remaining three days of each week, mice remained in abstinence with access to food and water *ad libitum*. Throughout the experiment, alcohol intake was measured each day as grams of EtOH per kilogram (g/kg) in each 2 and 4 h sessions. Thirty minutes after the last 4-h session (session 16), submandibular blood was extracted to determine blood alcohol concentrations (plasmatic EtOH, mg/dL) using a commercial Ethanol assay kit (Sigma-Aldrich, Madrid, Spain, Ref: MAK076).

### Synbiotic treatment administration

After 4 weeks of EtOH drinking, young mice (PD57) started chronic oral treatment with a SYN or its vehicle (VEH; H2O) for 3 weeks with free access during 24 h. The experimental groups were the following: H2O-VEH (*n* = 9), H2O-SYN (*n* = 10), EtOH-VEH (*n* = 10), and EtOH-SYN (*n* = 10). The SYN mixture was prepared fresh every day in drinking water and contained probiotics (1 × 10^9^ colony-forming units), including *(Lactobacillus rhamnosus, Lactobacillus acidophilus, Lactobacillus casei, Lactobacillus plantarum, Lactobacillus salivarius, Lactobacillus paracasei, Bifidobacterium bifidum, Bifidobacterium lactis, Bifidobacterium infantis, Bifidobacterium longum, and Bifidobacterium breve)* (Now Foods Probiotic-10, Bloomingdale, IL, USA), and the prebiotic inulin. We chose this particular synbiotic mixture based on previous studies in our laboratory using a model of alcohol-related addictive behavior in adult mice,^25^ and in a Down syndrome model of cognitive impairment^33^. Two different batches of mice underwent the same experimental conditions with 6 months of separation. The first batch included 5 mice H2O-VEH, 5 mice EtOH-VEH, 5 mice H2O-SYN and 5 mice EtOH-SYN. The second batch included 5 mice H2O-VEH, 5 mice EtOH-VEH, 4 mice H2O-SYN and 5 mice EtOH-SYN.

### Behavioral procedures

Behavioral testing started after three weeks of SYN treatment and lasted 2 weeks (PD78–94). All tests were conducted in mice treated with H2O-VEH (*n* = 10), H2O-SYN (*n* = 9), ETOH-VEH (*n* = 10) and ETOH-SYN (*n* = 10) by an observer blind to the experimental conditions. Behavioral tests were administered in order from the least to the most anxiogenic, based on the rationale that this approach helps ensure the validity and reliability of the experimental outcomes by minimizing potential carryover effects from anxiety-inducing environments. The battery of behavioral tests was conducted in the following sequence: Reference memory (1 day of test) − 1 day of washout – Novel object recognition (3 days of habituation, training, test) − 2 days of washout – Affective state discrimination (1 day of test) – Sociability/social novelty (1 day of test)- 1 day of washout – Marble burying (1 day of test) − 1 day of washout – Open field (1 day of test) − 1 day of washout – Tail suspension (1 day of test). All animals began the behavioral assessment at PD78 and tissue collection was performed at PD94, one day after completion of the tail suspension test.

#### Anxiety-like behavior

Anxiety-like behavior was measured with the open field, and marble-burying test. For the open field test, mice were placed in a square open field box (40x40cm) with a defined, unmarked center square. The center was illuminated with a 120-lux lamp. Mice were placed in the corner and were allowed to explore the box freely throughout the experiment, which lasted 10 minutes. Time spent moving around the center was measured to evaluate anxiety-like behavior. The marble burying test was performed using 10 marbles arranged in a grid pattern for a 30-minute test. Marbles were considered buried when at least two-thirds (approximately 75%) of their surface was covered by bedding as previously described.^25^

#### Depressive-like behavior

Despair-like behavior was measured with the tail suspension test. During this test, mice were suspended by their tails with tape from a bar 30 cm above the floor for 6 min. In the last 4 min, the time that mice spent as immobile was measured in seconds.

#### Social cognition

Social cognition was tested first in a three-chamber maze (20 cm length ×40 cm width ×22 cm height) as previously described.^34,35^ Before the assessment, mice were allowed to habituate to the maze for 5 min. To test sociability, an unfamiliar juvenile male mouse was placed in a wire containment box in one of the lateral chambers of the maze, and an identical empty box was placed in the opposite chamber. The walls separating the center of the maze with the lateral chambers were removed to begin the assessment. The mouse in the center was allowed to explore the maze for 10 minutes. The sociability index was calculated by measuring the difference between the time exploring the unfamiliar mouse and the time exploring the empty box, divided by the total time exploring. Subsequently, social novelty was tested by placing a different novel mouse in the wire containment box that was previously empty. Mice were allowed to freely explore for 10 min again. The social novelty index was calculated by subtracting the time exploring the familiar mouse from the time exploring the novel mouse, divided by the time exploring both mice. A sociability and social novelty index over zero indicate good social cognitive functioning in mice.

Then, the affective discrimination (ASD) test was performed as previously described.^36^ Mice were placed in a cage with two cylindrical wire containers and a cage divider between them to cover the reciprocal view between the wire containers while allowing the tested mice to observe both containers. One of the containers hosted a stressed mouse, while the other contained a neutral mouse. Stressed mice were subjected to the restrained tube test for 15 minutes to induce mild physiological stress as previously described.^37^ The tested mouse was allowed to freely explore and sniff both mice. The ASD index was calculated as a difference between time spent sniffing the stressed and the neutral mouse divided by the total time spent exploring both mice. An ASD index greater than 0 indicates that the mouse spent more time interacting with the distressed animal compared to the neutral one. This is interpreted as better performance in affective state discrimination.

#### Non-social cognition

Nonsocial cognitive functions included short-term and long-term memory, which were evaluated with the spatial reference memory and novel object recognition tests, respectively. Spatial reference memory was tested in an enclosed Y maze as previously described.^38^ First, during the training session mice were allowed to explore the Y maze with one of the arms, defined as the novel arm, closed off. Later in the testing session, the wall that closed the novel arm was removed and mice were allowed to explore the entire maze for 5 minutes. Time spent exploring the novel arm was measured. Higher time in the novel arm indicates better reference memory, while no discrimination between the arms of the Y maze indicates reference memory impairment.

For the novel object recognition test, a black Plexiglas maze with two corridors set at a 90° angle (Panlab, Spain) was used. This test consists of three phases, one each 24 h (habituation, training, and testing). On the 1st day, mice were habituated to the empty maze for 9 min. On the 2nd day, mice were trained in the maze for 9 min, but this time two identical objects were presented, one on each arm of the maze. On the 3rd day, mice were tested in the maze for 9 min, but one of the familiar objects was replaced by a novel object, and the total time spent exploring each object (novel and familiar) was recorded. A discrimination index was calculated as the time spent exploring the novel object minus the time exploring the familiar object divided by the total time exploring both objects. High discrimination index indicates greater memory retention and better long-term memory function.

### Sample collection

To evaluate the effects of alcohol consumption during adolescence on gut microbiota abundance and diversity we collected feces from mice after 4 weeks from H2O (*n* = 5) and EtOH (*n* = 5), and for SCFA quantification from H2O (*n* = 20) and EtOH (*n* = 20) mice at age PD57. To evaluate the effects of SYN treatment, fecal samples were collected 24 h after the last behavioral test at PD94 following 3 weeks of SYN and behavioral assessment for microbiota 16S rRNA metagenomic sequencing analysis from H2O-VEH (*n* = 8), H2O-SYN (*n* = 7), ETOH-VEH (*n* = 8) and ETOH-SYN (*n* = 8) groups, and for SCFA quantification from H2O-VEH (*n* = 9), H2O-SYN (*n* = 8), ETOH-VEH (*n* = 10) and ETOH-SYN (*n* = 10) groups. Brain samples from the PFC and HPC were collected from mice at PD 94 in the H2O-VEH (*n* = 10), H2O-SYN (*n* = 9), ETOH-VEH (*n* = 10) and ETOH-SYN (*n* = 10) groups. All samples were weighed and stored immediately at − 80°C until analysis.

### Library preparation and sequencing

To study the effects of alcohol drinking during adolescence and SYN treatment on gut microbiota diversity and composition, a total of 41 fecal samples from mice (*n* = 10 at PD57 and *n* = 31 at PD94) were processed for 16S ribosomal RNA subunit gene (16S rRNA) metagenomic sequencing analysis. DNA was extracted using the MagMAX CORE Nucleic Acid Purification Kit 500RXN (Thermo Fisher, Austin, USA) as instructed. Negative control of the DNA extraction as well as a positive Mock Community control (Zymobiomics Microbial Community DNA Standard, ZymoResearch, Irvine, CA, USA) for library preparation quality control was used. Samples were amplified targeting rRNA16S variable regions V3 and V4 with specific primers (16S Forward Primer 5‘TCGTCGGCAGCGTCAGATGTGTATAAGAGACAGCCTACGGGNGGCWGCAG 3,’ 16S Reverse Primer 5‘GTCTCGTGGGCTCGGAGATGTGTATAAGAGACAGGACTACHVGGGTATCTAATCC 3’). Amplicon PCR was performed in 10 μl final volume with 0.2 μM primer concentration, starting at denaturation at 95°C for 3 minutes with 25 cycles of amplification (95°C for 30 seconds, 55°C for 30 seconds, 72°C for 20 seconds) completed with elongation at 72°C for 5 minutes. The products underwent a purification from free primers and primer dimer species with AMPure XP beads (Beckman Coulter, Nyon, Switzerland). Indexing PCR was performed to attach full-length adapters for multiplex sequencing with Nextera XT v2 Index Kit (Illumina, San Diego, USA). 5 μl of the first PCR purified product underwent a second PCR with Nextera XT v2 adaptor primers in a final volume of 30 μl using the same PCR mix and protocol as described above during 8 cycles. 25 μl of the index PCR product were purified with SequalPrep normalization kit (Invitrogen, ThermoFisher Scientific, Waltham, MA, USA). Libraries were eluted in 20 μl final volume and pooled for sequencing. The final pool was quantified by qPCR using the Kapa library quantification kit for Illumina Platforms (Kapa Biosystems, SigmaAldrich, Saint Louis, MO, USA) on an ABI 7900HT real-time cycler (Applied Biosystems, ThermoFisher Scientific, Waltham, MA, USA). The Illumina Miseq (San Diego, USA) sequencing 300 × 2 approach was used with 15% of PhIX control libraries added to increase the diversity of the sequenced sample. Negative controls included sample collection buffer, DNA extraction, and PCR amplification steps, PRC products after both PCR steps were visualized by electrophoresis gel (1.5% agarose) with SYBR Safe (ThermoFisher Scientific, Waltham, MA, USA). No visible bands were observed.

### Amplicon sequence analysis

Raw demultiplexed forward and reverse reads were processed by following protocols using QIIME2 version 2019.4 as previously described.^39^ DADA2 software package was used for primer trimming, quality filtering, denoising, pair- end merging and phylotype calling with the amplicon correction protocol.^40^ Phylogeny assessment was done with multiple sequence alignment MAFFT method^41^ and the product alignments were used for FastTree phylogenetic tree and relationship computing.^42^ Taxonomic assignment of phylotypes was performed using a Bayesian Classifier trained with Silva database version 138 (99% OTUs full-length sequences) by a previously described method.^43^ Phylotype data was used to calculate observed OTU and evenness as measures of alpha diversity. Moreover, phylotype and phylogenetic data was used to calculate the metrics for beta diversity which include Bray Curtis, Jaccard, Weighted Unifrac and Unweighted Unifrac.

### Quantification of targeted metabolites in feces and brain

To quantify SCFA (propionic, butyric, valeric and isovaleric acids) in feces, fecal samples were homogenized with milli-Q water and the supernatant was analyzed using a liquid chromatography tandem mass spectrometry (LC-MS/MS) procedure adapted from a previously described method for urine samples.^44^ The samples went through a derivatization reaction where a solution of *o*-benzylhydroxylamine and *N-(3-dimethylaminopropyl)-N′-*ethylcarbodiimide hydrochloride reacts with the carboxylic acid groups of SCFA. After a liquid-liquid extraction, evaporation of the organic phase and reconstitution in milli-Q water and methanol (1:1) solution, 1 µL of the extract was injected into the LC-MS/MS system.

To quantify GABA, glutamate (Glu), glutamine (Gln) and BHB in the mPFC and HPC, samples (ca. 25 mg) were manually homogenized with 600 µL of a 2:1 mixture of acetonitrile:water with 0.1% formic acid using a glass homogenizer. The homogenates were then centrifuged at 14,500 rpm and 4°C for 5 min, and the supernatant was decanted into a clean Eppendorf tube and stored at −80°C until analysis. Homogenates were analyzed according to two previously reported methods.^44,45^

For analysis of neurotransmitters, an aliquot of 10 μl of brain homogenate was diluted 1:100 with ultrapure water. 10 µl of the diluted extract were transferred into a glass tube and spiked with 50 μl of the labeled internal standard mixture. Then, 130 μl of acetonitrile was added, and, after centrifugation (5 min; 145000 rpm), the supernatant was transferred to a micro vial and 5 µL were directly injected into the liquid chromatography-tandem mass spectrometry (LC-MS/MS) system.

For the analysis of BHB an aliquot of 20 μl of HPC or mPFC homogenates was transferred into a glass tube, was spiked with 30 μl of the labeled internal standard mixture, and was derivatized using 100 μl of a mixture of o-benzylhydroxylamine (1 M in acetonitrile:water 2:1) and *N*-(3-dimethylaminopropyl)-*N′-*ethylcarbodiimide (1 M in water:pyridine:HCl, 16:1.6:1; pH 5.0); at room temperature for 60 min, with continuous gentle mixing. After derivatization, 1 mL of ultrapure water was added, and the derivatized analytes were extracted with 4 mL of ethyl acetate. The organic layer was transferred into a glass tube and dried under a nitrogen stream ( < 15 psi) in a water bath set at 40°C. The extracts were reconstituted with 300 µL of methanol:water 1:1 and transferred to a micro vial for LC-MS/MS analysis. The injection volume was 10 µL.

For all measurements, LC-MS/MS system consisted on an Acquity I-Class UPLC system (Waters Associates) for the chromatographic separation coupled to a triple quadrupole (Xevo TQ-S micro) mass spectrometer provided with an orthogonal Z-spray-electrospray interface (ESI) (Waters Associates, Milford, MA, USA). Analytes were determined by a Selected Reaction Monitoring (SRM) method by acquiring two transitions for each compound, with the most specific one used for quantitation. Calibration curves were constructed by 1/x weighted least squares linear regression of the ratios between the peak areas of the compounds and their corresponding labeled internal standard. Besides the concentration of the analytes, ratios between selected metabolites were also considered to evaluate not only the concentrations of neurotransmitters, but also their formation and degradation. MassLynx software V4.1 and TargetLynx XS were used for data acquisition and processing.

### Statistical analysis

The effects of alcohol drinking during adolescence compared to water intake on alpha diversity, the relative abundance of microbiota taxa and fecal SCFA concentrations were analyzed with Welch’s Student t-test, and the Grubbs’ test was applied to identify statistical outliers, using a significance level of *p* < 0.05. The effects of SYN treatment on behavioral parameters, microbiota alpha diversity and abundance, and fecal SCFA concentrations were analyzed with two-way ANOVAs with alcohol (H2O and EtOH) and SYN (VEH and SYN) as between-subject factors. Fisher’s least significant differences test was used as a post-hoc when the two-way ANOVA showed interactions, and a significance was set at *p* < 0.05. To test the hypotheses that alcohol exposure would induce gut dysbiosis and induce associated changes in behavior and brain metabolites (based on our previous work in adult mice:),^25^ we performed Spearman correlation analyses between targeted metabolomics concentrations and behavioral parameters, relative abundance of gut microbiota, and fecal SCFA concentrations. The behavioral parameters included in the analysis were: depressive-like behavior (tail suspension test), social cognition (sociability, social novelty, and affective state discrimination), and nonsocial cognitive function (reference memory and long-term novel object recognition memory). The gut microbiota taxa included were: phyla *Firmicutes*, *Proteobacteria*, and *Actinobacteria*; families *Anaerovoracaceae*, *Clostridia UCG-014, Lactobacillaceae*, *RF39*, *Erysipelotrichaceae*, *Rhodospirillales* uncultured family and *Deferribacteraceae*; *species Enterorhabdus uncultured bacterium*, *Lactobacillus johnsonii* and *RF39* uncultured bacterium. The species *Lactobacillus johnsonii* and *RF39* uncultured bacterium were excluded from the analyses due to near zero values in the relative abundance of the control group. Fecal SCFA concentrations included butyric, isovaleric, propionic, and valeric acids. We acknowledge that conducting multiple correlation analyses increases the risk of false positives; however, given the hypothesis-driven nature of these tests and the limited sample size, we opted not to apply formal multiple testing corrections such as FDR, in order to preserve sensitivity to detect biologically meaningful associations. Missing values in metabolomic variables were imputed with the mean or median (based on data normality) of the available values, stratified by the treatment group. A minimum of 80% valid values were required to keep and impute each variable. The median absolute deviation (MAD) method was used to remove extreme outliers, with a threshold of 5 MADs from the median to ensure robustness. All metabolomic variables were log10-transformed. Data preprocessing and analyses were conducted using R software version 4.3.2. Graphics software included GraphPad Prism version 8.0.1 for Windows (San Diego, USA). Beta diversity ecological distance matrices were used to calculate and plot principal coordinates analysis (PCoA) with R software package version 3.6.0. Ecological distances between groups were compared with the Analysis of Similarity (ANOSIM) test. To estimate significant differences between ecological distances from samples after SYN treatment, the Permanova test was used with a significance set at *p < 0.05. BiodiversityR version 2.11–1, PMCMR version 4.3, RVAideMemoire version 0.9–7 and vegan version 2.5–5 packages were used.

## Results

### Changes in gut microbiota diversity and SCFA induced by alcohol drinking during adolescence

**Figure 1.**
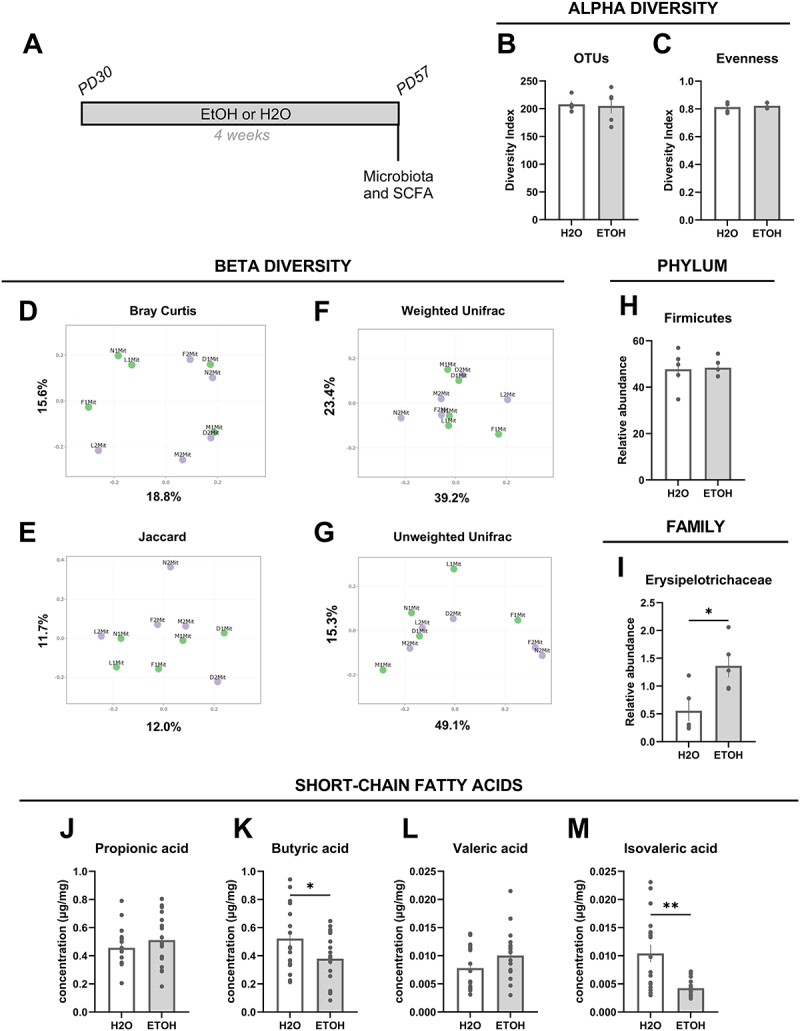
Alcohol-related alterations in gut microbiota and fecal short-chain fatty acids. (A) Schematic diagram of the experimental procedures followed. One group of adolescent mice (PD30) (*N* = 20) consumed alcohol (EtOH, grey bars) for 4 weeks following a “drinking in the dark” (DID) protocol until adulthood (PD57), and another consumed water (H2O, white bars) for 4 weeks (*N* = 20). Fecal samples were collected at PD57 from all mice. No significant EtOH-related changes were observed for alpha diversity in observed taxonomic units (OTU) (B) or evenness (C). Principal component analysis (PCoA) for microbial community beta diversity in fecal samples in mice exposed to H2O (purple round symbols) or ETOH (green round symbols) during adolescence: ecological distances for (D) Bray Curtis, (E) Jaccard, (F) unweighted, and (G) weighted Unifrac indexes. Although there were no significant differences between groups for the phylum *Firmicutes* (H), the relative abundance of a family belonging to this phylum, namely *Erysipelotrichaceae, was* significantly increased in mice drinking EtOH compared to those exposed to H2O (I). The fecal concentrations of the short-chain fatty acids butyric (K) and isovaleric (M) acids were significantly decreased in mice after 4 weeks of voluntary sub-binge alcohol drinking compared to mice exposed only to H2O. No significant differences between groups were observed in propionic (J) and valeric (L) acid concentrations. Values are mean ± SEM, statistical analysis by Welch’s t-test, *p < 0.05, **p < 0.01.

Fecal samples were collected after the DID protocol at PD57 (Figure 1(A)). Each week, adolescent mice were exposed to 20% alcohol solution 3 days a week during 2 h and for 4 h on the fourth day. On the days that alcohol was available, mice voluntarily consumed an average of 3 g/kg of alcohol (days 1–3, 2 h availability), and increased to 5 g/kg on day 4 (4 h availability) (Supplementary Figure S1A). In the last session of alcohol drinking (session 16), plasmatic alcohol concentrations reached 0.05 g/dl (Supplementary Figure S1B). Although this BAC is below the standard NIAAA levels considered for binge drinking (0.08 g/dl), our methodological approach was based on the well-established limited-access DID paradigm originally described by Rhodes et al.^26^ which has been widely adopted in preclinical research as a model for voluntary ethanol consumption that approximates binge-like intake. Thus, we consider that the procedure we used induced voluntary sub-binge alcohol drinking. Linear regression analysis revealed a positive correlation between alcohol intake (g/kg) and plasmatic alcohol (mg/dl) concentrations (*r=*0.61, *p<*0.01) (Supplementary Figure S1C).

Alpha and beta diversity were analyzed following the DID protocol in mice exposed to H2O or to alcohol. Alcohol drinking during adolescence had no significant effect on alpha diversity OTU or evenness indexes in the gut microbiota of adult mice (Figure 1(B,C)). Likewise, no significant differences were detected in calculated ecological distances (Figure 1(D-G)), indicating that alcohol drinking during adolescence has no effect on gut microbiota beta diversity in adult mice. On the other hand, alcohol consumption during adolescence caused a significant increase in the relative abundance of the *Erysipelotrichaceae* (*p* < 0.05) (Figure 1(I)) a microbial family belonging to the *Firmicutes* phylum (Figure 1(H)), and known to be involved in metabolic and inflammatory disorders ^46^. No significant alcohol-related changes were observed in other taxonomic groups present in adult mice fecal samples. Notably, quantification of SCFA in fecal samples indicated that alcohol caused a significant decrease in the fecal concentrations of butyric (*p* < 0.05) (Figure 1(K)) and isovaleric (*p<*0.01) (Figure 1(M)) acids. No significant changes were observed in fecal propionic (Figure 1(J)) and valeric (Figure 1(L)) acid concentrations.

### Synbiotic treatment modulates delayed behavioral alterations in mice exposed to alcohol drinking during adolescence

**Figure 2.**
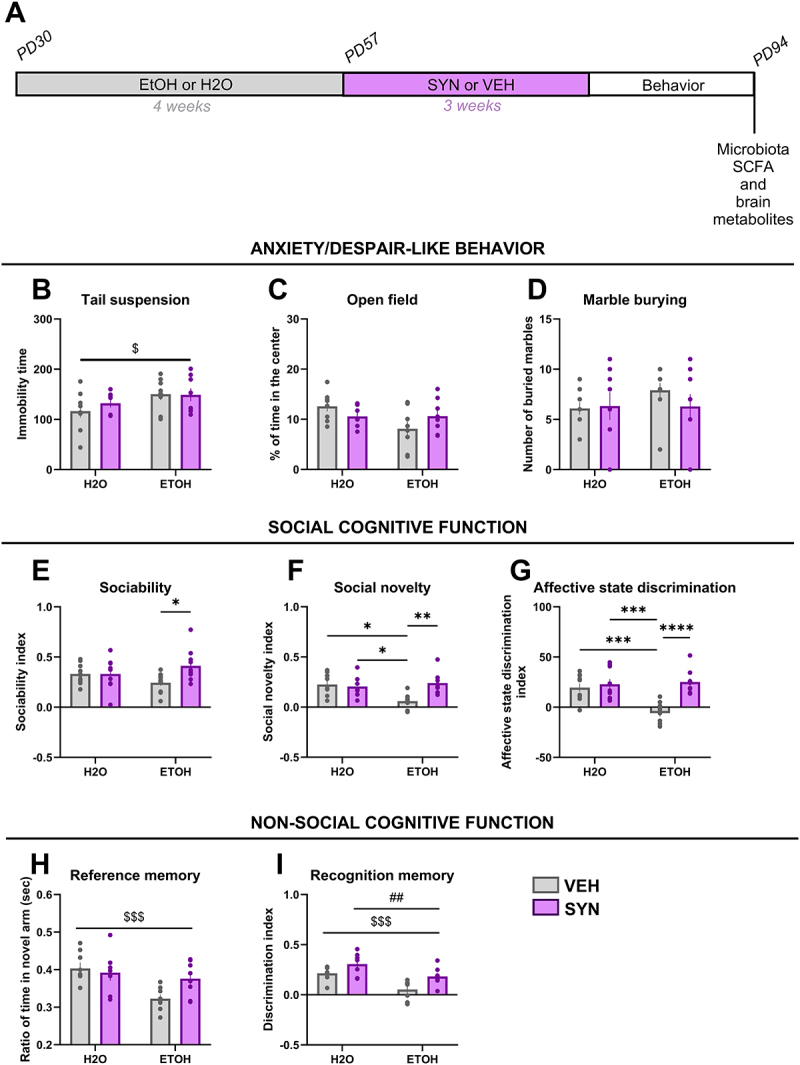
Behavioral effects of voluntary sub-binge alcohol drinking during adolescence and modulation by SYN treatment. (A) Schematic diagram of the experimental procedures followed in the study. After consuming either alcohol (EtOH) or water (H2O) from PD30 to PD57, each group was separated into two groups and administered either synbiotic (SYN) treatment or vehicle (VEH: water) for 3 weeks. Behavioral tests were performed at PD80 and continued for 1–2 weeks. Brain and fecal samples were collected at the end of the experiment. Mice were tested in different behavioral paradigms. (B) a significant main effect of EtOH on immobility time in the tail suspension test was observed. No significant changes were observed in % of time in the center of an open field (C) or in the number of buried marbles (D). A significant interaction between factors was observed in the sociability index (E), social novelty index (F), and affective state discrimination index (G), with post-hoc test showing significant differences between mice exposed to EtOH-VEH and H2O-VEH and between mice exposed to EtOH-SYN and EtOH-VEH. A significant main effect of alcohol on reference memory (H) and a significant main effect of alcohol and SYN on novel object recognition (I) were observed. Values are mean ± SEM, statistical analysis by two-way ANOVA followed by a post-hoc (main effect of EtOH: ^$^*p* < 0.05, ^$$$^*p* < 0.001); (main effect of SYN: ^##^*p* < 0.01); (*p < 0.05, **p < 0.01, ***p < 0.01).

We postulated that treatment with a SYN would provide beneficial effects on gut microbiota and SFCA concentrations counteracting those changes induced by alcohol drinking, and preventing potential delayed behavioral alterations through microbiota-gut-brain axis interactions.^24^ Thus, mice previously exposed to EtOH or water were administered SYN or VEH solutions during 3 weeks followed by behavioral testing. Fecal and brain samples were collected at the end of the procedures at PD94 (Figure 2(A)). The results showed a significant increase in immobility time in the tail suspension test irrespective of treatment with SYN or VEH (Figure 2(B)) (main effect of EtOH [F(1,27) = 3.667; *p* < 0.05). No significant effects of EtOH drinking or SYN treatment were observed for anxiety-like behavior in the open field or marble burying tests (Figures. 2(C,D)). A significant interaction between factors was observed for the sociability index (Figure 2(E)) [interaction: F(1,35) = 4.111; *p* < 0.05], and post-hoc comparisons revealed a significant difference between the group treated with EtOH-water and the one treated with EtOH-SYN (*p* < 0.05). Social novelty was significantly decreased in the EtOH-VEH group compared to the H2O-VEH group (*p* < 0.05), and SYN treatment significantly reversed this effect (*p* < 0.001) [interaction: F(1,28) = 7.878; *p* < 0.01] (Figure 2(F)). Similar results were obtained for affective state discrimination (Figure 2(G)), where EtOH significantly decreased this performance compared to the H2O-VEH group (*p* < 0.01), and SYN treatment significantly rescued this effect (*p* < 0.01) [interaction: F(1,35) = 11.68; *p* < 0.01]. For nonsocial cognitive function, a significant decrease in reference memory (Figure 2(H)) was found in the groups exposed to EtOH irrespective of treatment with SYN or VEH (main effect of alcohol: [F(1,27) = 8.823; *p* < 0.01]. Novel object recognition memory (Figure 2(I)) was significantly decreased in EtOH exposed groups compared to control groups (main effect of alcohol [F(1,27) = 18.07; *p* < 0.001], and this effect was reversed by SYN supplementation (main effect of synbiotic [F(1,27) = 11.10; *p* < 0.01].

### Synbiotic treatment modulates delayed changes in gut microbiota in mice exposed to alcohol drinking during adolescence

**Figure 3.**
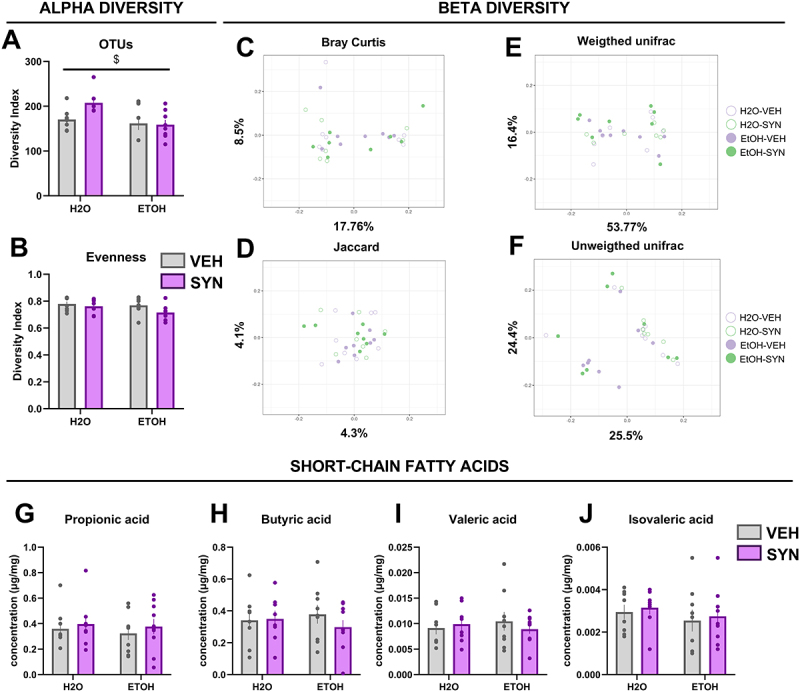
Changes in gut microbiota alpha and beta diversity and fecal SCFA concentrations induced by alcohol exposure during adolescence and modulation by SYN treatment. Adolescent mice were exposed to water (H2O) or alcohol (ETOH) during adolescence, and then received 3 weeks of treatment with vehicle (VEH, gray bars) or synbiotic (SYN, magenta bars). (A) a significant main effect of EtOH was observed in the diversity index of observed taxonomic units (OTU). No significant differences between groups were observed in evenness (B). Principal component analysis (PCoA) for microbial beta diversity in fecal samples for mice exposed to EtOH-VEH (filled purple circles), EtOH-SYN (filled green circles), H2O-VEH (empty purple circles), H2O-SYN (empty green circles): ecological distances for (C) Bray Curtis, (D) Jaccard, (E) unweighted, and (F) weighted Unifrac indexes. No significant differences in fecal concentrations of propionic (G), butyric (H), valeric (I), and isovaleric (J) acids were observed between groups. Values are mean ± SEM, statistical analysis by two-way ANOVA followed by a post-hoc test (main effect of EtOH, ^$^*p* < 0.05).

In terms of alpha diversity, a significant decrease was found in the OTU index (Figure 3A) for the groups that were exposed to EtOH during adolescence compared to control groups (main effect of EtOH [F(1,27) = 6.334; *p* < 0.05], but no significant main effect of SYN treatment or significant interaction between these two factors were observed. No significant changes were observed in the alpha diversity evenness index (Figure 3(B)), or in beta diversity calculated ecological distances (Figure 3(C-F)). Notably, the decrease in the concentrations of fecal butyric or isovaleric acids observed in young adult mice exposed to ethanol during adolescence did not persist until PD94 (Figure 3(G-J)). Thus, no significant effects of EtOH or SYN were found on the concentrations of SCFA in older mice.

**Figure 4.**
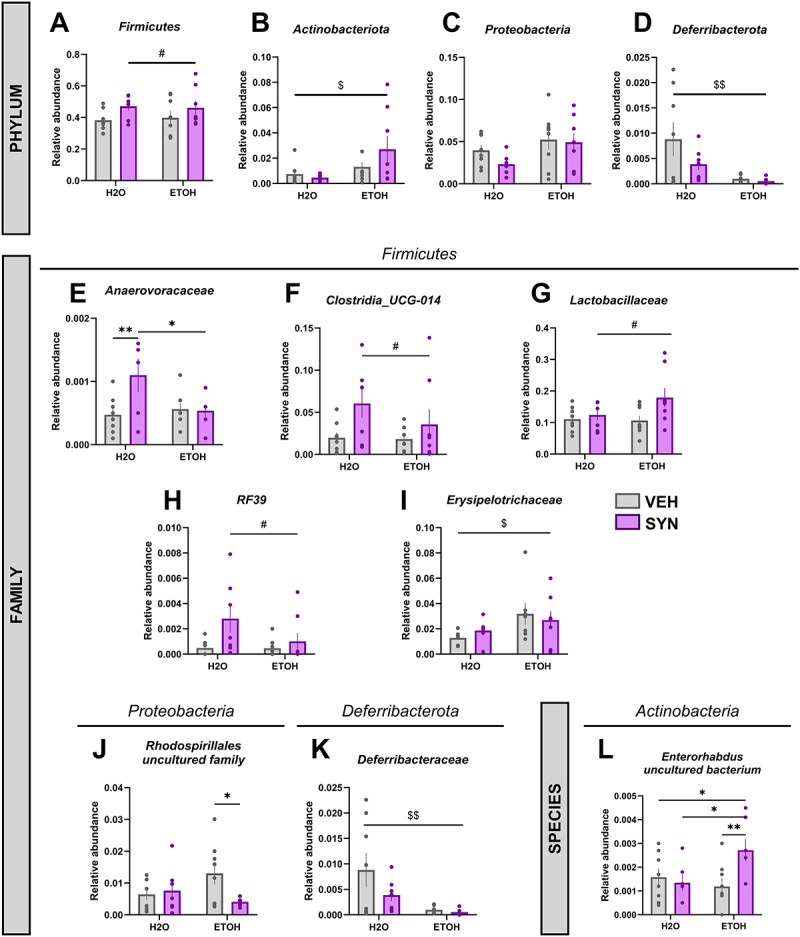
Changes in gut microbiota abundance induced by alcohol exposure during adolescence and modulation by SYN treatment. Adolescent mice were exposed to water (H2O) or alcohol (ETOH) during adolescence, and then received 3 weeks treatment with vehicle (VEH, gray bars) or synbiotic (SYN, magenta bars). A main effect of SYN treatment on the relative abundance of the *Firmicutes* (A) was observed. A significant main effect of EtOH on the relative abundance of the *Actinobacteria* (B) and *Deferribacterota* (D) was observed. For the members of Firmicutes phylum, an interaction was observed between factors in the *Anaerovoraceae* family (E). A significant main effect of synbiotic treatment was observed in *Clostridia UCG-014* (F), *Lactobacillaceae* (G) and *RF39* (H) families. A significant main effect of EtOH on the relative abundance of the *Erysipelotrichaceae* family (I) was observed. For the *Proteobacteria* phylum, in the uncultured family of *rhodospirillales* (J) a significant interaction between factors was observed. For the *Deferribacterota* phylum, a significant main effect of EtOH on the relative abundance of the family *Deferribacteraceae* (K) was observed. For the Actinobacteria phylum, in the *Enterorhabdus uncultured bacterium* family (L), a significant interaction between factors was observed. Values are mean ± SEM, statistical analysis by two-way ANOVA followed by a post-hoc. (main effect of EtOH: ^$^*p* < 0.05, ^$$^*p* < 0.01); (main effect of SYN: ^#^*p* < 0.05); (*p < 0.05, **p < 0.01).

However, several changes in the relative abundance of gut microbiota were observed in adult mice exposed to alcohol drinking during adolescence following SYN treatment. SYN treatment significantly increased the relative abundance of *Firmicutes* (Figure 4(A)) in mice exposed to H2O and in mice exposed to alcohol during adolescence (significant main effect of SYN, *p* < 0.05). Alcohol drinking in adolescence significantly increased the relative abundance of the *Actinobacteria* (*p* < 0.05) independently of SYN treatment. (Figure 4(B)). In contrast, alcohol drinking in adolescence significantly decreased the relative abundance of *Deferribacterota* (*p* < 0.01) (Figure 4(D)). No significant main effects of alcohol, SYN or significant interaction between factors were observed for *Proteobacteria* (Figure 4(C)).

In terms of the members of the *Firmicutes phylum*, a significant interaction between SYN treatment and alcohol drinking was detected in the relative abundance of *Anaerovoracaceae* family [F(1,27) = 4.776, *p* < 0.05] (Figure 4(E)), but no significant main effects were observed. A post-hoc analysis revealed significant differences between the H2O-SYN and H2O-VEH groups (*p<*0.01), and H2O-SYN and EtOH-SYN groups (*p* < 0.05). These results suggest that SYN treatment can increase the relative abundance of *Anaerovoracaceae* family in mice exposed to H2O while failing to produce the same effect in mice exposed to alcohol during adolescence. In addition, a significant main effect of SYN treatment was observed in the *Clostridia UCG-014* family (*p* < 0.05) (Figure 4(F)) where SYN treatment increased the relative abundance of this family in both mice exposed to H2O and alcohol. A main effect of SYN was also detected in the *Lactobacillaceae* family (*p* < 0.05) (Figure 4(G)), where SYN treatment increased the relative abundance of this family mostly in mice exposed to alcohol during adolescence. A main effect of SYN was revealed for the *RF39* family (*p* < 0.05) (Figure 4(H)) but no significant main effect of alcohol or significant interaction between factors were revealed. Notably, SYN treatment increased the relative abundance of *RF39* in groups exposed to both H2O and alcohol during adolescence. Interestingly, the effect of alcohol on the relative abundance of the *Erysipelotrichaceae* was also observed in adult mice (*p* < 0.05) (Figure 4(I)), moreover, this effect was driven by the relative abundance in the EtOH-VEH group.

In terms of the members of the *Proteobacteria* phylum, a significant interaction was observed between SYN treatment and alcohol drinking during adolescence in an uncultured family of the *Rhodospirillales* order [F(1,27) = 4.513, *p* < 0.05] (Figure 4(J)). A post-hoc analysis revealed that SYN treatment significantly decreased (*p* < 0.05) the relative abundance of this microbial family only in mice exposed to alcohol during adolescence, but not in those exposed to H2O.

For the *Deferribacterota phylum*, a significant main effect of alcohol was revealed in the relative abundance of *Deferribacteraceae* (*p* < 0.01) (Figure 4(K)) family, where alcohol drinking during adolescence caused a significant decrease in *Deferribacteraceae*, relative abundance independently of SYN treatment.

For the *Actinobacteria* phylum, a significant interaction was observed between SYN treatment and alcohol drinking during adolescence for the relative abundance of the species *Enterorhabdus uncultured bacterium* [F(1,27) = 5.040, *p* < 0.05] (Figure 4(L)). A post-hoc analysis revealed that SYN treatment significantly increased (*p* < 0.01) the relative abundance of this microbial species only in mice exposed to alcohol during adolescence, but not in those exposed to H2O.

### Synbiotic treatment modulates delayed effects in brain metabolites of mice exposed to alcohol drinking during adolescence

**Figure 5.**
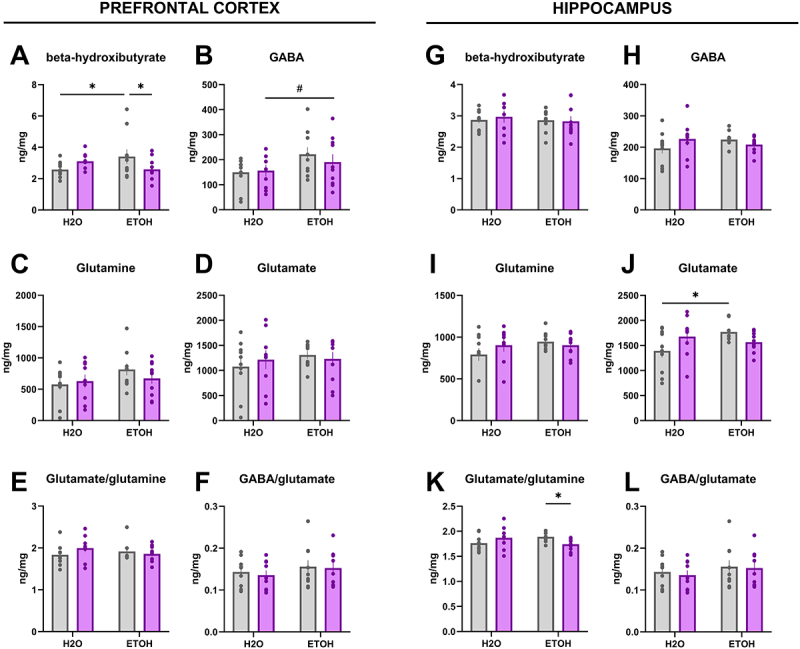
Effects of alcohol exposure during adolescence on brain metabolite concentrations and modulation by SYN treatment. Adolescent mice were exposed to water (H2O) or alcohol (ETOH) during adolescence, and then received 3 weeks of treatment with vehicle (VEH, gray bars) or synbiotic (SYN, magenta bars). (A) a significant interaction between factors was observed for prefrontal β-hydroxybutyrate concentrations, where EtOH increased its levels and SYN countered this effect. Moreover, a significant main effect of SYN was observed in the prefrontal concentrations of GABA (B). On the other hand, hippocampal glutamate (J) was increased by EtOH in animals receiving VEH but not SYN, whereas the Glu/Gln ratio (K) was decreased by SYN. Values are mean ± SEM, statistical analysis by two-way ANOVA followed by a post-hoc. (main effect of SYN: ^#^*p* < 0.05); (*p < 0.05).

In the PFC, BHB concentrations were increased in the EtOH-VEH group as compared to the H2O-VEH group (*p* < 0.05), and this effect was abrogated in the group treated with EtOH-SYN (*p* < 0.05) (interaction effect [F(1,35) = 5.63; *p* < 0.05] (Figure 5(A)). In addition, a significant main effect of SYN was observed for GABA concentrations [F(1,35) = 4.14; *p* < 0.05] (Figure 5(B)). No significant main effects of EtOH or SYN or interaction between these factors were observed for glutamate concentrations or its metabolism (Glu/Gln ratio) in the PFC (Figures. 5(C-F)). In the HPC, glutamate concentrations were increased in the group treated with EtOH-VEH as compared to the H2O-VEH group (*p* < 0.05), but not in the group receiving EtOH-SYN [interaction effect: F(1,33) = 5.17; *p* < 0.05] (Figure 5(J)). Moreover, a significant interaction was observed for the Glu/Gln ratio [F(1,33) = 5.38; *p* < 0.05], where SYN treatment significantly decreased this ratio only in the group treated with EtOH (*p* < 0.05) (Figure 5(K)). No significant main effects of EtOH or SYN or interaction between these factors were observed for BHB concentrations (5 G) or for the ratio GABA/Glu (5 L) in the HPC.

### Correlations between brain metabolite concentrations, behavioral tests and fecal SCFA

**Figure 6.**
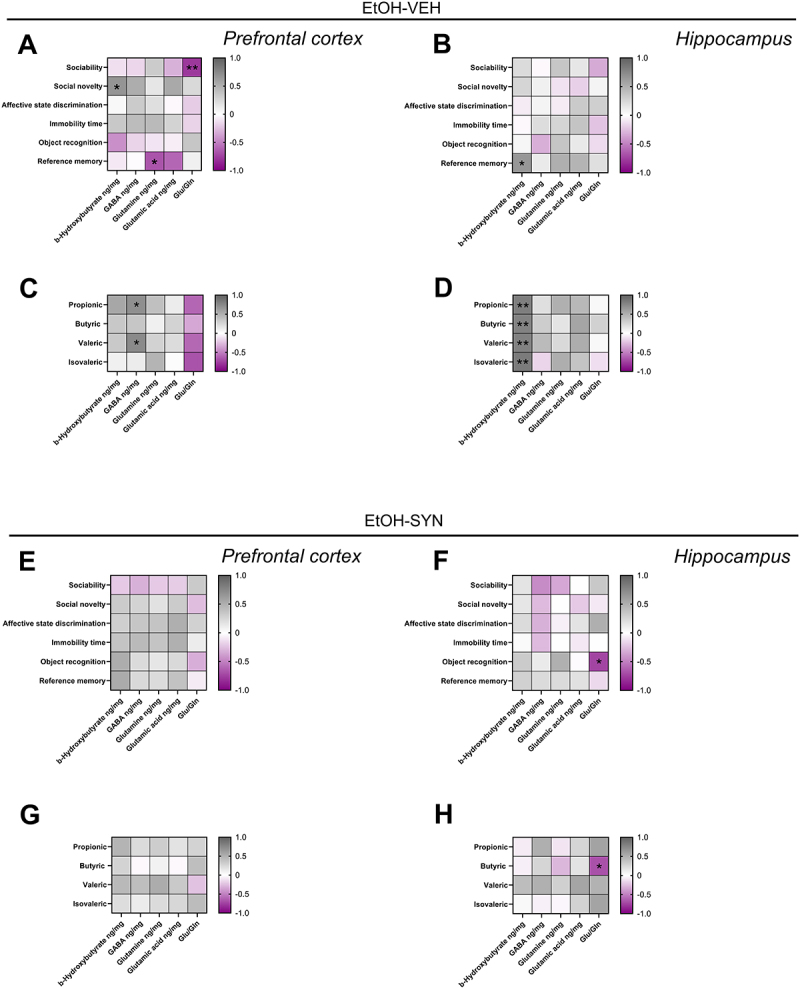
Spearman correlations between behavioral performance and brain metabolite changes and between changes in fecal SCFA and brain metabolite concentrations in mice exposed to EtOH-VEH and EtOH-SYN. In the EtOH-VEH group, the sociability index negatively correlated with the Glu/Gln ratio in the PFC. Social novelty positively correlated with PFC BHB, and reference memory showed a negative correlation with PFC Gln (A). Reference memory positively correlated with BHB in the HPC (B). GABA in the PFC correlated positively with propionic and valeric acids (C). BHB in the HPC correlated positively with propionic, butyric, valeric, and isovaleric acids (D). In the EtOH-SYN group, novel object recognition (F) performance and butyric acid (H) correlated negatively with Glu/Gln in the HPC. In the EtOH-SYN group, no significant correlations were found between brain metabolites and behavioral tests (E) or SCFA (G). Gln: Glutamine; Glu: glutamate. The dependent variables in the behavioral tests are: sociability index, social novelty index, affective state discrimination index, immobility time in the tail suspension test, novel object discrimination index for object recognition test and ratio of time in novel arm for reference memory test. The color-coded side bar represents the R of Spearman (from −1 to 1), and significant correlations are marked with asterisks (*p < 0.05, **p < 0.01).

For metabolites determined in the PFC in the EtOH-VEH group, a significant negative correlation was observed between the ratio of Glu/Gln and the sociability index (*R* = −0.80, *p* < 0.01). A positive correlation was found between the concentrations of BHB and social novelty (*R* = 0.70, *p* < 0.05), and a negative correlation between the concentrations of glutamine and reference memory (*R* = −0.70, *p* < 0.05) (Figure 6(A)). In addition, GABA concentrations in the PFC correlated positively with the fecal concentrations of propionic (*R* = 0.70, *p* < 0.05) and valeric acids (*R* = 0.66, *p* < 0.05) (Figure 6(C)). All of these correlations were abolished in the EtOH-SYN group (Figure 6(E,G)). For metabolites determined in the HPC in the EtOH-VEH group a positive correlation was observed between BHB and reference memory (*R* = 0.64, *p* < 0.05) (Figure 6(B)). In addition, BHB also correlated positively with the fecal concentrations of propionic (*R* = 0.78, *p* < 0.01), butyric (*R* = 0.70, *p* < 0.01), valeric (*R* = 0.68, *p* < 0.01), and isovaleric (*R* = 0.70, *p* < 0.01) acids (Figure 6(D)). In the EtOH-SYN group, all these correlations were abolished (Figure 6(F,H)). In this group, novel object recognition performance correlated negatively with the ratio of Glu/Gln (*R* = −0.733, *p* < 0.05) (Figure 6(F)). Similarly, butyric acid correlated negatively with the ratio Glu/Gln (*R* = −0.70, *p* < 0.05) (6 H).

### Correlations between gut microbiota bacterial populations, behavioral tests, fecal SCFA and brain metabolites

**Figure 7.**
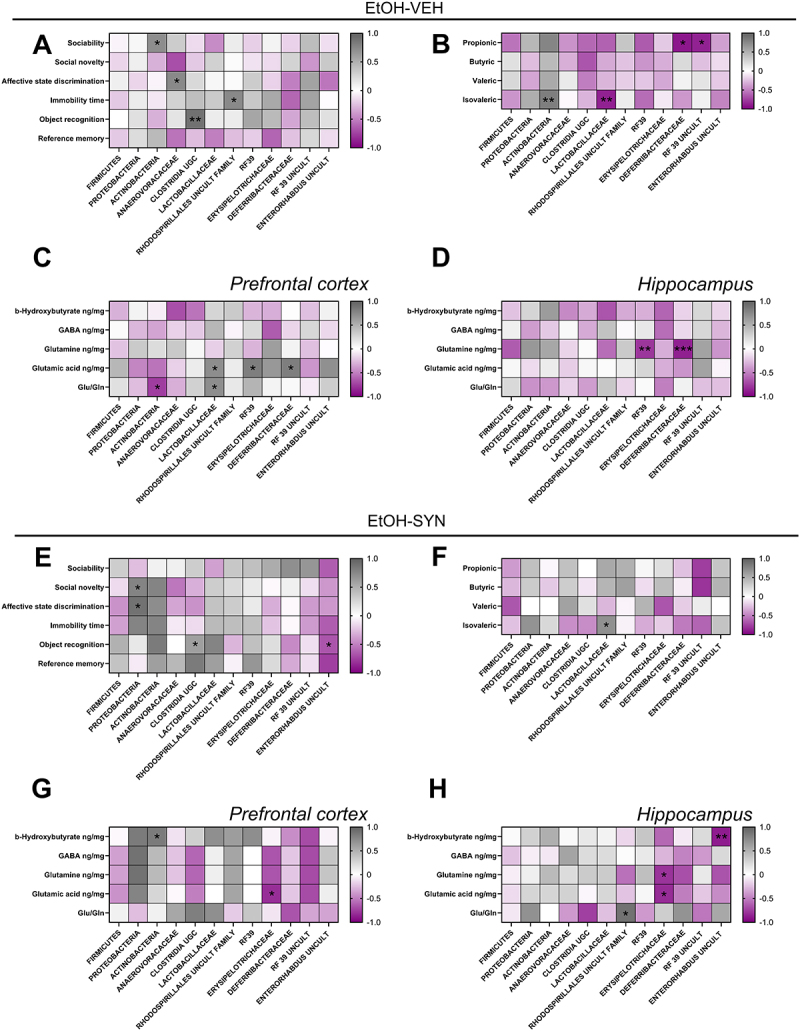
Spearman correlates between behavioral performance and gut microbiota abundance changes and fecal SCFA, between brain metabolites and gut microbiota changes, and between gut microbiota and SCFA in mice exposed to EtOH-VEH and EtOH-SYN. In the EtOH-VEH group, the phylum *Actinobacteria* correlated positively with sociability index, and the *Anaerovoracocaceae* family correlated positively with affective state discrimination. The *Rhodospirillales* family correlated positively with immobility time, and *Clostridia* correlated positively with the novel object recognition index (A). The dependent variables in the behavioral tests are: sociability index, social novelty index, affective state discrimination index, immobility time in the tail suspension test, novel object discrimination index for object recognition test and ratio of time in novel arm for reference memory test. In addition, *actinobacteria* correlated positively and *Lactobacillaceae* correlated negatively with isovaleric acid, while *Deferribacteraceae* correlated negatively and *RF39 uncultured bacterium* correlated negatively with propionic acid (B). In the PFC, *Actinobacteria* correlated negatively with Glu/Gln whereas *Lactobacillaceae* correlated positively with glutamic acid and Glu/Gln. Moreover, *Deferribacteraceae* and *RF39* correlated positively with glutamic acid (C). In the HPC, *Deferribacteraceae* correlated negatively and *RF39* correlated positively with glutamine (D). In the EtOH-SYN group, the phylum *Proteobacteria* correlated positively with affective discrimination, social novelty and with immobility time. Also, object recognition correlated positively with *Clostridia UGC* and negatively with *Enterorhabdus* (E) and *Lactobacillaceae* correlated positively with isovaleric acid (F). In the PFC, BHB correlated positively with *Actinobacteria* and *Eryspelotrichaceae* correlated negatively with glutamic acid (G). In contrast, BHB correlated negatively with *Enterorhabdus uncultured, Eryspelotrichaceae* correlated negatively with glutamine and glutamic acid and *Rhodospirillales* correlated positively with Glu/Gln (H).The color-coded side bar represents the R of Spearman (from −1 to 1), and significant correlations are marked with asterisks (*p < 0.05, **p < 0.01, ***p < 0.001).

In the EtOH-water group, the phylum *Actinobacteria* correlated positively with sociability index (*R* = 0.71, *p* < 0.05). In terms of gut microbial families, *Anaerovoracocaceae* correlated positively with affective state discrimination (*R* = 0.70, *p* < 0.05); *Rhodospirillales* correlated positively with immobility time (*R* = 0.70, *p* < 0.05), and *Clostridia* correlated positively with the novel object recognition index (*R* = 0.90, *p* < 0.01) (Figure 7(A)). In addition, *Actinobacteria* showed a positive correlation with isovaleric acid (*R* = 0.89, *p* < 0.01), while *Lactobacillaceae* correlated negatively with isovaleric acid (*R* = −0.9, *p* < 0.01). Both *Deferribacteraceae* (*R* = −0.98, *p* < 0.01) and *RF 39 uncultured bacterium* (*R* = −0.94, *p* < 0.05) correlated negatively with propionic acid (Figure 7(B)). For metabolites determined in the PFC in the EtOH-VEH group, glutamate concentrations positively correlate with *Deferribacteraceae* (*R* = 0.77, *p* < 0.05) and with *RF39* (*R* = 0.78, *p* < 0.05). A significant negative correlation was observed between the ratio of Glu/Gln and *Actinobacteria* (*R* = −0.76, *p* < 0.05). *Lactobacillaceae* correlated positively with the ratio Glu/Gln (*R* = 0.71, *p* < 0.05), and with glutamate concentrations (*R* = 0.71, *p* < 0.05) (Figure 7(C)). For metabolites determined in the HPC in the EtOH-VEH group, glutamine correlated negatively with *RF39* (*R* = −0.80, *p* < 0.05), and with *Deferribacteraceae* (*R* = −0.95, *p* < 0.001) (Figure 7(D)).

In the EtOH-SYN group, the phylum *Proteobacteria* correlated positively with affective discrimination index (*R* = 0.90, *p* < 0.05), with social novelty index (*R* = 0.8, *p* < 0.05) and immobility time (*R* = 0.80, *p* < 0.05); object recognition correlated positively with Clostridia UGC (*R* = 0.75, *p* < 0.05) and negatively with *Enterorhabdus* (*R* = −0.78, *p* < 0.05) (Figure 7(E)). Finally, *Lactobacillaceae* correlated positively with isovaleric acid (*R* = 0.75, *p* < 0.05) (F).

For metabolites determined in the PFC in the EtOH-SYN group, *Actinobacteria* correlated positively with BHB (*R* = 0.9, *p* < 0.05), and *Erysipelotrichaceae* correlated negatively with glutamate (*R* = −0.9, *p* < 0.05) (Figure 7(G)). For metabolites determined in the HPC in the EtOH-SYN group, *Rhodospirillales* correlated positively with the Glu/Gln ratio (*R* = 0.9, *p* < 0.05); *Erysipelotrichaceae* correlated negatively with glutamine (*R* = −0.8, *p* < 0.05), and with glutamate (*R* = −0.8, *p* < 0.05), and *Enterorhabdus* correlated negatively with BHB (*R* = −0.9, *p* < 0.01) (Figure 7(H)).

### Correlations between behavioral tests and fecal SCFA

In the EtOH-VEH group, no significant correlations were observed between behavioral tests and SCFA, while in the EtOH-SYN group, social novelty correlated negatively with valeric acid (*R* = −0.64, *p* < 0.05) (Supp. Figure 2(B)).

## Discussion

We evaluated whether intermittent alcohol drinking during adolescence induces gut dysbiosis and if these alterations impact brain metabolism and behavioral function later in life. Our data indicate that voluntary sub-binge alcohol drinking during adolescence induces selective changes in gut microbiota, namely an increase in abundance of the microbial family *Erysipelotrichaceae*, and a decrease in the fecal concentrations of butyric and isovaleric acids. Interestingly, while these changes are restored at a later age, delayed alterations in other types of gut bacteria appear associated with behavioral and brain metabolite profile disruptions. Notably, the administration of a SYN treatment restores the long-term alcohol-induced changes observed in gut microbiota, and exerts beneficial effects on alcohol-induced social and nonsocial cognitive impairments and brain metabolite concentration changes.

To unveil the effects of alcohol drinking during adolescence on gut microbiota we examined fecal samples in mice exposed to either water or alcohol after a four-week DID protocol. For gut microbiota diversity, we report that adolescent alcohol drinking had no immediate impact either on alpha or on beta diversity. These findings are supported by previous similar results in adolescent rats after intragastric intermittent alcohol exposure.^15^ The lack of effect observed in our study may be attributed to homeostatic mechanisms that restore overall bacterial diversity during the four weeks of alcohol drinking. However, we found significant changes in the relative abundance of one bacterial family, namely *Erysipelotrichaceae*. Our finding of higher concentrations of *Erysipelotrichaceae* in mice that consumed alcohol in adolescence compared to those consuming water is consistent with previous reports of increased relative abundance of the *Erysipelotrichales* order in mice after the Lieber-DeCarli ethanol diet,^47^ and in mouse models of acute colitis.^48^ In addition, *Erysipelotrichaceae* has been associated in humans with irritable bowel syndrome,^49^ and TNF-α driven Crohn’s Disease,^50^ suggesting that alcohol drinking during adolescence may be closely linked to microbiota alterations observed in inflammatory diseases. Analysis of fecal SCFA concentrations revealed that mice drinking alcohol during adolescence have lower butyric and isovaleric acid concentrations compared to control mice. SCFA are byproducts of fiber and protein metabolism of gut microbiota important for maintaining gut homeostasis by modulating host immunity through their anti-inflammatory properties and by regulating microbial growth.^51,52^ Therefore, SCFA alterations could be interpreted as a readout of gut dysbiosis. Interestingly, lower concentrations of isovaleric acid have been linked with depression in humans,^53^ and low fecal butyric acid concentrations have been associated with gut barrier dysfunction in Parkinson disease patients,^31^ and with cognitive dysfunction in patients reporting mild cognitive impairments.^32^ These findings are consistent with the increasing amount of evidence pointing to SCFA as mediators of the microbiota-gut-brain axis crosstalk,^54^ thus modulating brain function and behavior.

Our next step was to investigate whether the administration of a SYN targeting gut dysbiosis would attenuate delayed behavioral alterations induced by alcohol drinking in adolescence. We found that alcohol induced a slight depressive-like phenotype in adulthood, which was not modulated by SYN, but it did not provoke clear anxiety-related behavior. The depressive-like behavior observed in our study is in line with previous evidence showing that a similar alcohol drinking protocol during adolescence produces delayed behavioral alterations in the forced swimming test.^55^ In that study, delayed anxiety-like behavior was also observed. The lack of significant results in terms of anxiety in our study may be explained by the fact that marble burying and the time spent in the center of an open field arena in mice are modulated by both innate exploratory curiosity and by anxiety.^56^ Hence, it may be possible that alcohol drinking during adolescence modulates both of these aspects of behavior in opposite ways, with no net effect. Our results are however in line with the findings in studies using chronic intermittent alcohol administration procedures in mice showing a persistent impairment in object recognition performance, with no spatial working memory impairments or anxiety-like behavior,^57^ and with other results showing a decrease in recognition memory performances and behavioral flexibility in both adult males and females subjected to a similar DID procedure during adolescence.^55^ Indeed, we found that alcohol drinking during adolescence provoked impairments in both reference and novel object recognition memory. Interestingly, we also observed delayed impairments in several aspects of social cognition. Delayed social novelty impairments were observed in line with what was found by Van Hees et al.^55^ In addition, sociability performance, as well as affective state discrimination were affected in adults. To our knowledge, this is the first study that reports an impairment in affective state discrimination caused by alcohol drinking in adolescence.

Notably, we found that treatment with a SYN for 3 weeks can override alcohol-related impairments in sociability, social novelty, affective state discrimination, and novel object recognition memory. The results showing a beneficial effect of the dietary supplement used in this study on novel object recognition memory deficits has been previously reported in adult mice exposed to a chronic intermittent protocol of alcohol drinking,^25^ and a recent study reports that that supplementation with the prebiotic fiber inulin, modulates gut microbiota and social alterations in alcohol use disorder patients.^58^ To our knowledge, however, we report for the first time the beneficial effects of a SYN treatment on delayed social cognition impairments in mice exposed to DID protocol in adolescence.

The beneficial effects of SYN treatment on alcohol-induced behavioral impairments were paralleled by changes in the relative abundance of specific gut microbial taxa. We found that the increase in the relative abundance of the *Rhodospirillales uncultured* family, and the decrease in the *Enterorhabdus uncultured species* induced by alcohol was abrogated by SYN administration. *Rhodospirillales* is an opportunistic bacterium reported to increase when host immunity is compromised,^59^ hence it is possible that *Rhodospirillales* may take advantage of alcohol-related altered gut homeostasis and proliferate, but SYN treatment reverts its abundance back to normal concentrations by increasing other beneficial bacteria. One of these beneficial bacteria may belong to the *Enterorhabdus* genus, which has been associated with antidepressant effects,^60^ probably by an action on tryptophan and kynurenine metabolism.^61^ Our results show that SYN treatment increases *Enterorhabdus uncultured species* in mice exposed to alcohol during adolescence. While we did not observe any effect of SYN on depressive-like behavior following alcohol drinking, these findings suggest that SYN has a beneficial effect on gut microbiota homeostasis by decreasing disease-related bacteria and increasing beneficial microbes. Consistently with this notion, SYN increased the relative abundance of the beneficial bacterial family *Lactobacillaceae* only in mice exposed to alcohol. On the other hand, we found that the persistent increase observed in the pro-inflammatory family *Erysipelotrichaceae* was not significantly reversed by SYN treatment.

**Figure 8.**
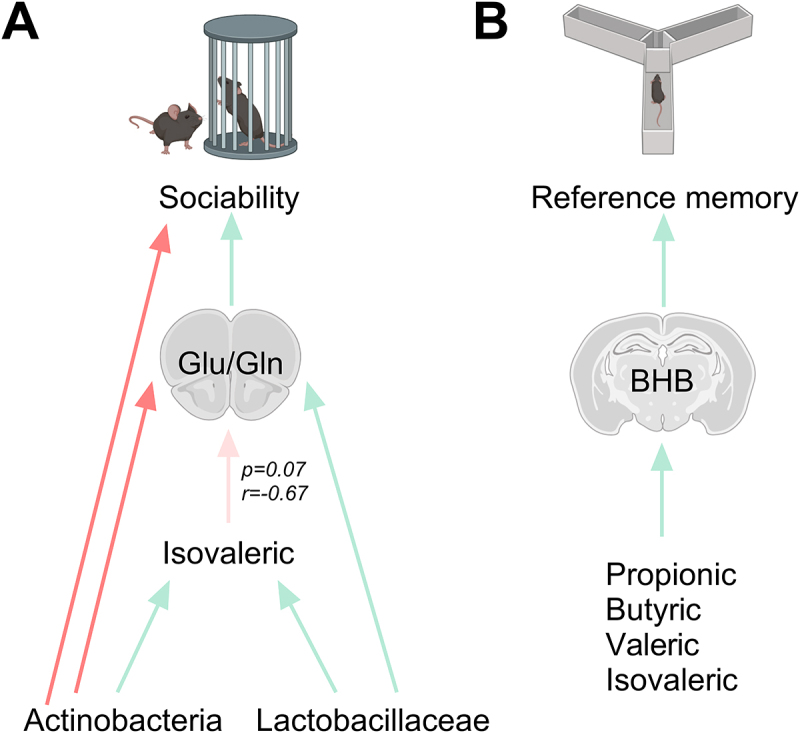
Schematic representation of the gut microbiota-PFC glutamate metabolism-sociability network (A) and the SCFA-HPC BHB-spatial memory relationship (B). Red arrows indicate Spearman positive correlation whereas green arrows indicate negative correlation.

Several reports indicate that a number of SCFA that enter the brain have modulatory effects on neurotransmitters such as glutamate and GABA (for a review, see).^54^ Therefore, the disturbances induced by alcohol drinking during adolescence may have caused changes in these neurotransmitters’ metabolism in the PFC and HPC, which would have ultimately resulted in delayed behavioral deficits. In this regard, several relevant associations were found between behavioral alterations, changes in gut SCFA, microbiota abundance, and brain metabolites in mice exposed to alcohol drinking during adolescence that were modulated by SYN treatment. For example, we identified a potential functional gut microbiota-PFC glutamate metabolism-sociability network relating both *Actinobacteria* and *Lactobacillaceae* to isovaleric acid, glutamate metabolism in the PFC and sociability index (see Figure 8(A)). In line with these findings, prior research has demonstrated that specific microbial metabolites can control the PFC’s glutamate and GABA concentrations (for a review, see).^62^ Moreover, it has been recently reported that maternally separated mice exhibiting social impairments, anxiety-like behavior and gut dysbiosis, also show alterations in the concentrations of Gln, Glu and GABA in the PFC.^63^ Notably, in that study, the administration of *Lactobacillus reuteri* ameliorates social dysfunction and restores PFC concentrations of these neurotransmitters by increasing the expression of intestinal amino acid transporters.^63^ On the other hand, we distinguished another correlation hub relating SCFAs (propionic, butyric, valeric and isovaleric acids) with BHB in the HPC and reference memory (see Figure 8(B)). Previous studies have reported that detoxified patients with alcohol use disorder, as well as mice transplanted with feces from alcohol use disorder patients show an association between low BHB plasma concentrations and social impairments (Leclercq et al., 2020). However, to our knowledge this is the first study showing an association between BHB concentrations in the HPC and gut microbiota metabolites (SCFAs) potentially impacting on nonsocial cognition. BHB is synthesized in the liver as the most important ketone body that provides energy especially during periods of fasting or caloric restriction. It can be transported to the brain via the monocarboxylate transporter MCT1, where it serves as an energy substrate for neurons and has a variety of properties that influence brain function.^64^ Importantly, all of these correlations were not present in the group of mice exposed to alcohol drinking and treated with SYN, supporting the contention that targeting gut dysbiosis with a SYN could modulate detrimental microbiota-gut-brain axis interactions and reverse behavioral alterations following alcohol drinking in adolescence.

In conclusion, this study provides correlative data related to the consequences of adolescent voluntary sub-binge alcohol drinking on behavior, and how they can be modulated by SYN supplementation possibly through optimizing microbiota-gut-brain axis interactions. One limitation of our study is the increased risk of false positives due to the number of correlations examined. While our analyses were guided by prior hypotheses, and correction methods were not applied to preserve statistical power, these findings should nonetheless be interpreted with caution and warrant further validation in larger cohorts. It should be addressed that the small number of subjects used in the gut microbiota analysis could have impeded the discovery of other potential significant effects of both alcohol and SYN. Additionally, this study was performed only in male mice, so further research is needed to elucidate sex-specific differences. Finally, we evaluated the efficacy of the SYN as a whole, rather than dissecting the contributions of individual elements. As such, our findings do not distinguish whether the effects are due to one or more probiotic strains, the prebiotic fiber, or synergistic interactions among them. Future studies should evaluate the individual and combined effects of each component. Even so, the findings can provide precedents for further research in alcohol-induced dysbiosis and ways to deal with the public health problem of adolescent drinking.

## Supplementary Material

Barrera_Conde_et_al_2025_Revised_Suppl_Figures.docx

## Data Availability

Raw data were generated at Hospital del Mar Research Institute. Derived data supporting the findings of this study are available at DOI:10.5281/zenodo.15595280.
