# Supplementary material for: Synbiotic intervention reverses alcohol drinking-induced cognitive deficits in adolescent male mice by modulating the microbiota-gut-brain axis
Source: Gut Microbes. 2025 Sep 1;17(1):2551104. doi: 10.1080/19490976.2025.2551104 (PMC12407653; doi:10.1080/19490976.2025.2551104)
Supplement: Barrera_Conde_et_al_2025_Revised_Suppl_Figures.docx [file KGMI_A_2551104_SM1875.docx]

Synbiotic Administration Modulates Microbiota-Gut-Brain Interplay in Binge Drinking-Induced Social and Cognitive Deficits in Adolescent Male Mice

Marta Barrera-Conde1, Elizaveta Korchevaya2,3, Elk Kossatz1, Emma Veza1, Mitona Pujadas1,4, Élida Alechaga5, Pau Nebot5, Oscar J. Pozo5, Rafael de la Torre1,2,4, Nieves Pizarro1,2 and Patricia Robledo1

1Integrative Pharmacology and Systems Neuroscience Research Group, Neuroscience Research Programme, Hospital del Mar Medical Research Institute, Barcelona, Spain.

2Department of Medicine and Life Sciences, University Pompeu Fabra, Barcelona, Spain.

3Research Programme on Biomedical Informatics (GRIB), Hospital del Mar Medical Research Institute, Barcelona, Spain.

4CIBER de Fisiopatología de la Obesidad y Nutrición (CIBERON), Instituto de Salud Carlos III, Madrid, 28029, Spain.

5Applied Metabolomics Research Group, Neuroscience Research Programme, Hospital del Mar Medical Research Institute, Barcelona, Spain.

Corresponding author:

Patricia Robledo, PhD

Integrative Pharmacology and Systems Neuroscience Research Group Hospital del Mar Research Institute

PRBB

Calle Dr. Aiguader 88 Barcelona 08003 SPAIN

[probledo@researchmar.net](mailto:probledo@researchmar.net) Tel: +34 93 316 0045

**Supplementary Figure 1**

**
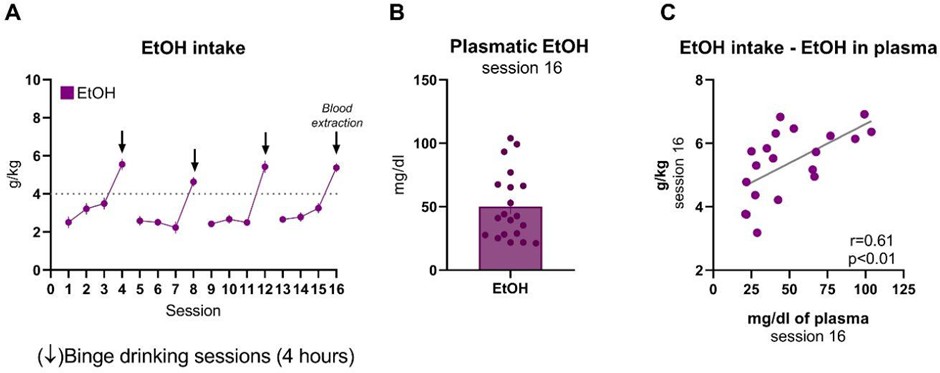
**

**Supplementary Figure 1.** (A) Alcohol (EtOH) intake (g/kg) during the 4 weekly sessions of drinking in the dark in adolescent mice. Arrows indicate binge drinking sessions with free 4 h access to EtOH (20%) (see Methods). (B) Plasmatic EtOH levels (mg/dl) were measured in the last binge session (session 16).

(C) Linear regression between EtOH intake in session 16 and plasmatic EtOH levels.

**Supplementary Figure 2**

**Sociability Social novelty**

**A**

**Affective state discrimination**

**Immobility time Object recognition Reference memory**

**Propionic**

**Butyric**

**Valeric**

# B

*

**Sociability Social novelty**

**Affective state discrimination**

**Immobility time Object recognition Reference memory**

**Propionic**

**Butyric**

**Valeric**

**Isovaleric**

1.0

0.5


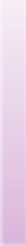


0

-0.5

-1.0

**Isovaleric**

1.0


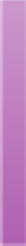


0.5

0

-0.5

-1.0

**Supplementary Figure 2. Spearman correlations between behavioral performance and fecal SCFA, in mice exposed to EtOH-VEH and EtOH-SYN.** No significant correlations were observed between behavioural performance and fecal SCFA concentrations in the EtOH-VEH group (A). In the EtOH-SYN group social novelty correlated negatively with valeric acid (B). The dependent variables in the behavioral tests are: sociability index, social novelty index, affective state discrimination index, immobility time in the tail suspension test, novel object discrimination index for object recognition test and ratio of time in novel arm for reference memory test. The color-coded side bar represents the R of Spearman (from -1 to 1), and significant correlations are marked with asterisks (*p<0.05).
